# Temporal Variation in Mechanical and Chemical Properties of Bamboo Decayed by *Schizophyllum commune* QP33

**DOI:** 10.3390/jof12030175

**Published:** 2026-02-28

**Authors:** Xinyi Guo, Xiaolong He, Xiaojiao An, Yaojie Sang, Chengjing Ren, Yuqin Luo, Yan Zhang, Xinxing Wu, Jun Qian, Hui Wang, Fangli Sun, Shuaibo Han

**Affiliations:** 1 School of Chemical and Materials Engineering, National Engineering & Technology Research Center of Wood-Based Resources Comprehensive Utilization, Zhejiang A&F University, Hangzhou 311300, China; 2023104022004@stu.zafu.edu.cn (X.G.); 2022604051005@stu.zafu.edu.cn (X.H.); axj624@stu.zafu.edu.cn (X.A.); 19858531306@163.com (Y.S.); renchengjing2004@163.com (C.R.); zhangy@iccas.ac.cn (Y.Z.); xinxingwu@zafu.edu.cn (X.W.); wanghui@zafu.edu.cn (H.W.); 2 Jiangshan Fenghe Home Furnishing Co., Ltd., Quzhou 324100, China; 13967034944@163.com; 3 Microbes and Insects Control Institute of Bio-Based Materials, Zhejiang A&F University, Hangzhou 311300, China; 4 Jiangshan Industry Innovation Research Institute of Door Industry and Whole House Customization, Quzhou 324100, China; junqian@zafu.edu.cn

**Keywords:** bamboo, *Schizophyllum commune*, biodegradation, mechanical properties, chemical composition, microstructure

## Abstract

As an important biomass material, bamboo is susceptible to fungal infection during use, leading to severe deterioration. The white-rot fungus *Schizophyllum commune* is one of the world’s most widely distributed fungi, which preferentially colonizes dead or senescent bamboo tissues. However, the mechanism of the influence of the *S. commune* infection on the mechanical and chemical properties of bamboo remains unexplored. This research systematically examined the temporal effects (0, 30, 60, and 90 days) of *S. commune* QP33 infection on bamboo’s mechanical properties and chemical composition using various characterization methods. Results showed that *S. commune* QP33 secreted key lignin-modifying enzymes (laccase and lignin peroxidase) and hemicellulases (xylanase). Mass loss of bamboo increased progressively with infection time, reaching 13.33% after 90 days. Decayed bamboo showed distinct mechanical deterioration patterns, including a sharp initial drop in bending strength and a continuous decline in tensile strength. Microstructural and chemical analyses revealed that the fungus preferentially degraded lignin and hemicellulose. This selective degradation led to cell wall delamination and pore formation, ultimately causing the observed macroscopic mechanical deterioration. Our study provides critical insights into the biodeterioration mechanism of bamboo by *S. commune* and offers valuable guidance for bamboo preservation and high-value utilization.

## 1. Introduction

Bamboos are a unique group of giant arborescent grasses, characterized by woody stems that grow from underground rhizomes. As one of the fastest-growing and most versatile plants on earth, it holds significant economic and cultural value, serving as a food source, construction material, and raw ingredient for multifunctional products. Bamboo is a natural composite material that shares wood’s excellent strength-to-weight ratio. This characteristic allows it to be widely used in construction, including houses, bridges, and furniture [1]. In rural regions rich in bamboo resources, it also serves as a primary load-bearing material for low-rise buildings [2]. As a natural hierarchical porous material, bamboo exhibits excellent mechanical properties along its fiber direction, including tensile and flexural strength. The unique structural characteristics of natural bamboo make it an ideal renewable resource for high-performance composite materials [3,4].

However, natural bamboo exhibits poor durability due to its rich internal nutrients such as starch and sugar, making it susceptible to microbial degradation in humid environments [5,6]. This leads to a decline in mechanical properties, significantly limiting its application in construction. Among the degrading agents, decaying fungi pose the most severe threat [7]. Research indicates that brown-rot fungi degrade bamboo at varying rates. For example, *Gloeophyllum trabeum* caused 25% mass loss in 4 months, whereas *Coniophora puteana* caused 11.2% over 6 months [8,9]. In contrast, white-rot fungi demonstrated stronger degradation effects. *Trametes versicolor* exhibited the highest decay rate (62.5% mass loss) [9,10]. Among soft-rot fungi, *Chaetomium globosum* induced moderate degradation (9.6% mass loss), whereas *Paecilomyces variotii* caused minimal damage (3.1% mass loss) [1,11]. Additional studies highlight the pronounced destructive potential of white-rot fungi in bamboo decay experiments [12,13].

As a globally widespread white-rot fungus, *Schizophyllum commune* (split gill mushroom) primarily colonizes and degrades hardwood and bamboo substrates on every continent except Antarctica [14,15]. It serves as a critical decomposer in lignocellulosic ecosystems due to its enzymatic capacity to degrade woody substrates [16,17]. Notably, it demonstrates exceptional colonization efficiency in bamboo ecosystems, penetrating bamboo through multifaceted pathways including transverse invasion via outer and inner culm cell walls, cross-sectional infiltration, and longitudinal progression through nodal ridges and vascular bundles [16,18,19]. Given its recognized degradative potential, Schmidt found that *S. commune* exhibited a mass loss of 15% in bamboo under specific experimental conditions [9]. Recently, *S. commune* has been widely used as one of the wood-decaying fungi in durability tests of bamboo or bamboo products, alongside *Trametes versicolor* and *Ganoderma lucidum* [11,20,21]. However, the impact of its decay on the mechanical properties and chemical composition of bamboo remains unclear, particularly the mechanistic progression of degradation over time. This lack of understanding limits the creation of effective preservation methods.

Therefore, this study aims to systematically investigate the degradation mechanism of *Schizophyllum commune* QP33 on bamboo by correlating its temporal degradation patterns (0, 30, 60, and 90 days) with the evolution of mechanical properties and chemical composition. The findings are expected to provide new insights for protecting bamboo resources and developing fungal-bamboo composite materials.

## 2. Materials and Methods

### 2.1. Isolation and Optical Microscopy of Schizophyllum commune QP33

The samples of decayed bamboo were prepared according to our previous study [6]. The central cross-sectional area of the bamboo segments was aseptically sampled by scraping to a depth of 1 mm with a sterilized knife. The scraped epidermis of bamboo was placed into sterile water and vortexed to achieve thorough mixing. Subsequently, the mixture was subjected to serial dilution with sterile water to obtain dilutions of different concentrations (10^−1^, 10^−2^, 10^−3^, 10^−4^, and 10^−5^). A 100 µL aliquot of each dilution was evenly spread on the surface of potato dextrose agar (PDA) plates, with three replicates per concentration. The plates were incubated in an incubator at 26–28 °C and 75% relative humidity for 3–5 days. After the colonies grew, the tip hypha picking method was used to select colonies with distinct morphologies, which were then transferred to fresh PDA medium for purification. The purified strains were inoculated onto slant medium and stored at 4 °C for subsequent use. Laccase production of all fungal isolates was screened on PDA medium supplemented with 0.04% guaiacol as substrate [22].

The fungal strain QP33, exhibiting pronounced reddish-brown zones in the PDA-guaiacol media, was chosen for further investigation. The micro morphology of strain QP33 was observed via an optical microscope (DW-100, Hangzhou DW Microbiology Co., Ltd., Hangzhou, Zhejiang, China). Strain QP33 was cultured on Potato Dextrose Agar (PDA) and incubated at an appropriate temperature for 5–6 days. During this incubation period, a sterile coverslip was inserted obliquely into the agar at the edge of the actively growing colony. The Petri dish was resealed and returned to the incubator for an additional 2–3 days to allow fungal growth along the coverslip surface. The coverslip was then carefully removed, and the adherent mycelium was stained with a drop of cotton blue staining solution [23]. After brief rinsing with deionized water, the sample was air-dried or flame-fixed gently over an alcohol lamp. Finally, the stained coverslip was mounted on a microscope slide for observation under an optical microscope.

### 2.2. Molecular Identification and Phylogenetic Analysis of Schizophyllum commune QP33

DNA extraction and PCR amplification were performed following the protocol established by Jović et al., with some modifications [14]. The internal transcribed spacer (ITS) region of the ribosomal RNA coding DNA was amplified by PCR using the primer set ITS1 (5′-TCCGTAGGTGAACCTGCGG-3′)/ITS4 (5′-TCCTCCGCTTATTGATATGC-3′). The completely extracted DNA functioned as a template. The following components were present in the 50 µL reaction mixture: 1 µL DNA template, 1 µL forward primer (ITS1, 10 µmol/L), 1 µL reverse primer (ITS4, 10 µmol/L), 25 µL 2 × T5 Super PCR Mix (Tsingke Biotechnology Co., Ltd., Hangzhou, China) and 22 µL ultrapure distilled water. The PCR procedure was established as follows: 5 min at 95 °C for initial denaturation, 30 cycles of denaturation (30 s at 95 °C), annealing (30 s at 55 °C) and elongation (60 s at 72 °C), and 10 min at 72 °C for final extension. Amplified PCR products were sequenced by Tsingke Biotechnology Co., Ltd. (Hangzhou, China). The resulting sequence representing the partial sequence of ITS region was compared for pairwise similarity using the online Basic Local Alignment Search Tool (BLAST, https://blast.ncbi.nlm.nih.gov/Blast.cgi, accessed on 15 April 2025). Phylogenetic analysis was conducted by collecting ITS gene sequences from the NCBI of closely related strains. Clustal W1.8 was used to align multiple sequences [24]. The Mega 11 software tool was used to rebuild phylogenetic trees using the maximum likelihood method [25,26]. To determine evolutionary distances, we used Kimura’s two-parameter model methodology for the aforementioned approaches [27]. The phylogenetic tree’s robustness was evaluated using a bootstrap analysis with 1000 replications.

### 2.3. Determination of Ligninolytic Enzymes Activity of Schizophyllum commune QP33

Mycelia of the fungus at the logarithmic growth phase were scraped from a PDA culture plate. The mycelia were mixed with an appropriate buffer at a 1:10 (*w*/*v*) ratio for extraction. The mixture was sonicated on ice using a Biosafer 1000 ultrasonic homogenizer (Biosafer, Hangzhou, China) and then centrifuged at 9500× *g*, 4 °C for 10 min using a GTR216C high-speed refrigerated centrifuge (Beijing Era Beili Centrifuge Co., Ltd., Beijing, China) [28]. The supernatant was harvested and maintained on ice for immediate enzyme activity analysis.

Laccase activity was determined based on the oxidation rate of ABTS (2,2′-azino-bis (3-ethylbenzothiazoline-6-sulfonic acid), Aladdin, Shanghai, China). The assay system consisted of 0.8 mL sodium acetate buffer (100 mmol/L, pH 4.5), 0.1 mL ABTS (0.5 mmol/L), and 0.1 mL enzyme extract. Enzyme activity (U/L) was defined as the amount that oxidized 1 nmol of ABTS per minute, using a molar extinction coefficient (ε_420_) of 36,000 L·mol^−1^·cm^−1^ [29]. The absorbance was measured using a C4S UV-Vis spectrophotometer (Shanghai Mapada Instruments Co., Ltd., Shanghai, China).

Lignin Peroxidase (LiP) activity was measured based on the oxidation of veratryl alcohol. The reaction system consisted of 0.6 mL of tartrate buffer (250 mmol/L, pH 3.0), 0.2 mL of veratryl alcohol solution (10 mmol/L, prepared from veratryl alcohol, Aladdin), 0.1 mL of appropriately diluted supernatant (10-fold), and 0.1 mL of hydrogen peroxide solution (10 mmol/L). One unit of enzyme activity (U/L) was defined as the amount of enzyme needed to oxidize 1 nmol of veratryl alcohol per minute. The molar extinction coefficient (ε_310_) for veratryl alcohol is 9300 L·mol^−1^·cm^−1^ [30,31].

The activity of Manganese Peroxidase (MnP) was assessed through the oxidation of guaiacol. The assay mixture comprised 0.6 mL of succinate buffer (50 mmol/L, pH 4.5), 0.1 mL of 10 mmol/L MnSO_4_ (Macklin, Shanghai, China), 0.1 mL of 10 mmol/L guaiacol (Aladdin, Shanghai, China), 0.1 mL of enzymatic supernatant, and 0.1 mL of 10 mmol/L H_2_O_2_ to initiate the reaction. Enzyme activity, expressed in U/L, was quantified as the amount needed to oxidize 1 nmol of guaiacol per minute. Calculation was based on the molar extinction coefficient of guaiacol at 465 nm (ε_465_ = 12,100 L·mol^−1^·cm^−1^) [28].

The activity for all enzymes was calculated using the following Formula (1):

(1) $$\mathrm{Enzyme}\;\mathrm{Activity}\left(U/L\right)=\frac{∆\mathrm{OD}}{\varepsilon \times b\times ∆t}\times V_{\mathrm{total}}/V_{\mathrm{enzyme}}\times 10^{9}$$

where:

ΔOD: change in absorbance during the reaction time

ε: molar extinction coefficient of the respective substrate (as specified above)

b: light path length of the cuvette (cm)

Δt: reaction time (min)

V_total_: total volume of the reaction mixture (mL)

V_enzyme_: volume of the enzyme solution used in the reaction (mL)

### 2.4. Determination of Hemicellulase Activities of Schizophyllum commune QP33

The activities of key hemicellulolytic enzymes were determined spectrophotometrically. The general procedure involved incubating a suitably diluted enzyme solution with a specific substrate under defined conditions (50 °C, pH 5.0), and monitoring the resulting product.

Xylanase activity was determined based on the release of reducing sugars from birchwood xylan. The assay mixture, containing 0.5 mL of appropriately diluted enzyme extract and 0.5 mL of 1% (*w*/*v*) birchwood xylan in 50 mM sodium citrate buffer (pH 5.0), was incubated at 50 °C for 30 min. The reaction was terminated by adding 1.5 mL of DNS reagent, followed by boiling for 10 min. After cooling, absorbance was recorded at 540 nm. Enzyme activity (U/mL) was defined as the amount of enzyme liberating 1 μmol of reducing sugar (as xylose equivalent) per minute under the assay conditions [32,33].

β-Xylosidase activity was measured using p-nitrophenyl-β-D-xylopyranoside (pNPX) as the substrate. A reaction mixture of 0.5 mL of 1 mM pNPX in 50 mM sodium citrate buffer (pH 5.0) and 0.5 mL of enzyme solution was incubated at 50 °C for 10 min. The reaction was terminated by the addition of 1.0 mL of cold 1 M sodium carbonate solution. Absorbance at 400 nm was used to quantify the amount of released p-nitrophenol (pNP). One unit of activity (U/mL) was stated as the amount of enzyme releasing 1 μmol of pNP per minute [34].

The following formula was used to determine the activity for all hemicellulases:

(2) $$\mathrm{Enzyme}\;\mathrm{Activity}\left(U/L\right)=(C\times V_{\mathrm{total}}\times D)/(T\times V_{\mathrm{enzyme}})$$

C: Concentration of the product (reducing sugar or pNP) determined from the standard curve (μmol/mL)

V_total_: Total volume of the reaction mixture (mL)

D: Dilution factor

T: Reaction time (min)

V_enzyme_: Volume of the enzyme solution used in the reaction (mL)

Note: Standard curves must be prepared using xylose (for xylanase) or p-nitrophenol (for β-xylosidase) under identical assay conditions.

### 2.5. Bamboo Sample Preparation and Fungal Cultivation

**Figure 1:**
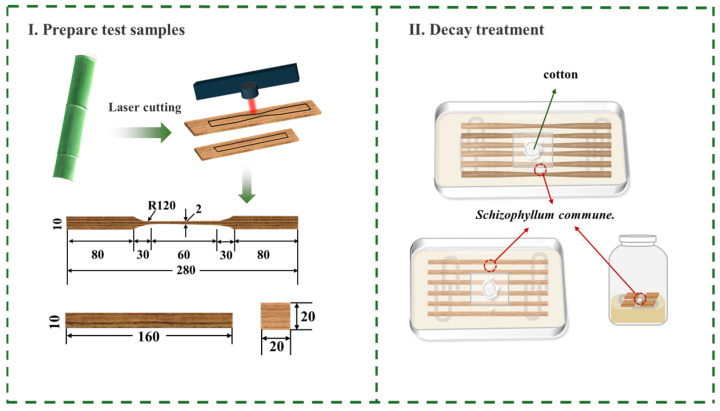
Experimental scheme of bamboo decay. (**I**) Preparation of standardized bamboo specimens by laser cutting. (**II**) Fungal decay treatment, showing inoculation and incubation of samples with S. commune QP33. Green arrows denote the specimen preparation steps, and red arrows indicate the fungal inoculation and decay pathway.

The bamboo (*Phyllostachys edulis*) samples for the decay experiment were collected from Lin’an, Zhejiang Province, China, and were prepared using a laser cutting machine (22 mm/s). Standard specimens were cut according to GB/T 15780-1995 [35] for tensile (280 × 10 × 5 mm^3^), three-point bending (160 × 10 × 5 mm^3^), and compression tests (20 × 20 × 5 mm^3^), as shown in Figure 1I. Following drying at 105 °C to constant weight and sterilization at 121 °C for 60 min, the specimens were maintained under sterile storage conditions.

The fungus *S. commune* QP33 was first cultured on PDA at 28 °C for 5–7 days, until the mycelium fully colonized the Petri dish. The PDA medium was prepared by dissolving PDA powder (Shanghai Haibo Microbial Technology Co., Ltd., Shanghai, China) in ultrapure water at a mass ratio of 46:1000 (powder: water). Approximately 1 cm depth of the medium was poured into high-temperature-resistant, food-grade plastic culture containers (dimensions: 325 × 223 × 101 mm and 190 × 190 × 116 mm). The lids were perforated and plugged with cotton balls for gas exchange. The containers were then sterilized by autoclaving for 20 min.

After sterilization, cooling, and solidification of the medium, four U-shaped glass rods were arranged in a cross pattern inside each container. Activated *S. commune* QP33 was inoculated into the culture box using a cork borer to transfer mycelial plugs from the pre-cultured PDA, as illustrated in Figure 1II. Bamboo strip specimens were then placed on the glass rods. The lids were securely sealed with parafilm to maintain a controlled environment.

The specimens were incubated under constant conditions of 28 °C and 75% relative humidity for experimental periods of 0 (control), 30, 60, and 90 days, with 12 replicates per treatment group. To prepare for subsequent analysis, the cultivated bamboo strips were harvested and oven-dried to a constant weight at 105 °C.

### 2.6. Mass Loss Measurement

Mycelium was stripped from the surface of bamboo strips and blocks infested for different days, dried in a gradient (60 °C for 2 h, 80 °C for 2 h, 105 °C for 4 h) to a constant weight, and weighed as m_1_. The rate of mass loss (ML) was determined according to Equation (3).

(3) $$\mathrm{ML}=\frac{m_{0}-m_{1}}{m_{0}}$$

### 2.7. Morphological Characterization of Fungus-Treated Bamboo

The morphological changes of bamboo specimens (including control, 30-day, 60-day, and 90-day fungal treatment groups) were characterized using scanning electron microscopy (SEM) and ultra-depth three-dimensional microscopy. Prior to observation, the specimens were precision-cut into blocks measuring 5 × 5 × 2 mm (L × W × H) using a sliding microtome and baked to achieve complete dryness. For SEM analysis, the dried blocks were mounted on specimen stubs using conductive adhesive, followed by vacuum sputter-coating with a gold layer to enhance conductivity. The surface morphology of the specimens was then examined using a scanning electron microscope (Quattro ESEM, Thermo Fisher Scientific, Waltham, MA, USA) operated at an accelerating voltage of 12.5 kV.

Simultaneously, the bamboo specimen after the mechanical property test was evaluated using an ultra-depth 3D microscope system (VHX-1000, KEYENCE Corp., Osaka, Japan). This system utilized an extended depth-of-field technique to generate high-resolution three-dimensional images, enabling precise measurement of the height and depth profiles of the fracture surface after the mechanical property test.

### 2.8. Mechanical Property Evaluation of Fungus-Treated Bamboo

**Figure 2:**
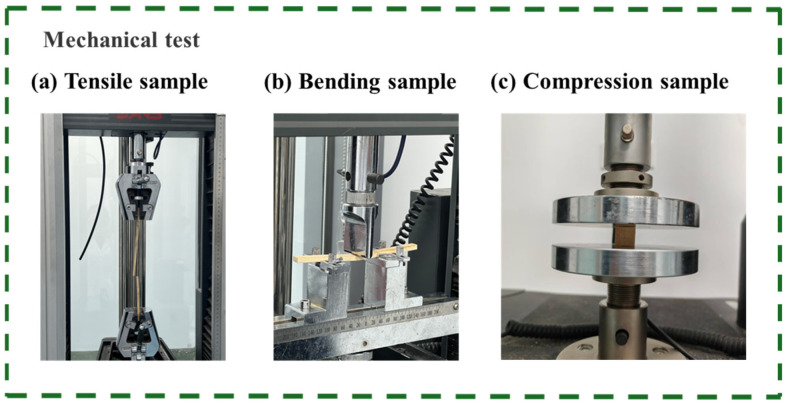
Schematic representation of the mechanical test setup.

As shown in Figure 2a, flexural strength tests were performed on a universal testing machine (CMT6104) using a three-point bending setup. Specimens measuring 160 × 10 × 5 mm^3^ were placed on two supports with a span of 120 mm. A load was applied at the midspan at a constant crosshead speed of 3 mm/min until failure. The maximum flexural strength (MOR) and modulus of elasticity (MOE) were calculated from the load-displacement data.

As shown in Figure 2b, tensile tests were conducted using the same universal testing machine (CMT6104). Specimens measuring 280 × 10 × 5 mm^3^ were clamped at both ends. A constant crosshead speed was applied to achieve specimen failure within 1 ± 0.5 min, and the maximum failure load was recorded.

As shown in Figure 2c, compressive strength was measured using a universal material testing machine (INSTRON 5960). Specimens (20 × 20 × 5 mm^3^) were placed at the center of the spherical sliding support, and a load was applied parallel to the fiber direction at a constant crosshead speed selected to cause failure within 1 ± 0.5 min. The failure load was recorded.

### 2.9. Analysis of Chemical Composition

Bamboo decayed by strain QP33 at various stages were ground into powder. Functional groups in the bamboo powders were characterized using a Fourier-transform infrared spectrometer (IRPrestige-21, Shimadzu Corp., Kyoto, Japan). Samples (200 mesh) were prepared by mixing with potassium bromide at a 1:50 ratio and pressing into pellets prior to scanning across the 400–4000 cm^−1^ range [36]. The composition and elemental changes of bamboo blocks before and after decay were determined via a K-Alpha X-ray photoelectron spectrometer (Thermo, USA). A monochromatic Al Kα excitation source was used, including an excitation energy of 1486.6 eV and an operating voltage of 12 kV [37].

XRD spectra of the various decayed bamboo powders were collected using an X-ray diffraction analyzer (Ultima IV, Rigaku Corp., Tokyo, Japan). The samples were uniformly spread on a quartz holder and scanned in reflectance mode using a horizontal goniometer over a 10–50° range with a step size of 0.05° and a scanning speed of 4° min^−1^ [38]. The relative crystallinity index (CrI) was subsequently calculated using Formula (4).

(4) $$\mathrm{CrI}\left(\%\right)=\frac{I_{002}-I_{\mathrm{am}}}{I_{002}}\times 100\%$$

where I_002_ is the diffraction intensity in the crystalline region, and I_am_ is the diffraction intensity in the amorphous region.

The D_002_ grain size was calculated using Scherrer’s Formula (5)

(5) $$D_{002}\;\left(\mathrm{nm}\right)=\frac{K-\lambda }{\mathrm{Bhkl}\;\times \;\cos\theta }$$

where λ is the wavelength of incident X-rays (0.154 nm), Bhkl is the half-width (radians) of the diffraction peak of the crystalline surface, K is a constant (taken as 0.9) and cos θ is the cosine of the diffraction angle.

The d_002_ crystal plane spacing was calculated using Bragg’s equation (Formula (6))

(6) $$d_{002}\left(\mathrm{nm}\right)=\frac{n\times \lambda }{2\times \sin\theta }$$

where λ is the wavelength of the incident X-rays (0.154 nm), n is the number of reflection levels (taken as 1) and sin θ is the sine of the diffraction angle.

### 2.10. Statistical Analysis

ANOVA analysis of the mechanical data using IBM SPSS Statistics 26 (IBM Corp., Armonk, NY, USA) to evaluate the effects of different groups on the bamboo material and the confidence level of the test. Differences with a *p*-value < 0.05 were regarded as statistically significant.

## 3. Results and Discussion

### 3.1. Morphological Biological Identification

**Figure 3:**
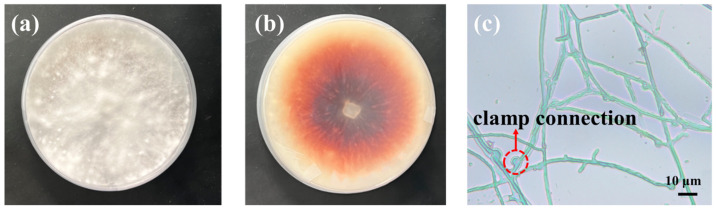
Morphological characteristics of *S commune* QP33. (**a**) Colony morphology on PDA medium after 7 days of incubation. (**b**) Oxidative coloration of guaiacol on PDA medium, indicating ligninolytic enzyme activity. (**c**) Hyphal morphology observed under light microscope (300×); arrows indicate typical clamp connections.

Strain QP33 was cultured on potato dextrose agar (PDA) medium supplemented with 0.04% guaiacol and incubated at 28 °C for 7 days to observe its colonial and hyphal morphology. The colony was cotton-like with a white, loose texture (Figure 3a). The addition of guaiacol to the PDA medium served as a rapid and effective phenotypic indicator for screening lignin-degrading fungi. The oxidative coloration (reddish-brown) surrounding the colony of strain QP33 provided direct visual evidence of extracellular ligninolytic enzyme activity (particularly laccase and/or peroxidases (Figure 3b). As shown in Figure 3c, the hyphae of strain QP33 formed dense network structures through clamp connections, a typical feature of basidiomycetous fungi. During the decay of bamboo, these hyphae may penetrate the interface between the fiber sheath and parenchyma cells, disrupting the gradient structure of vascular bundles and interrupting stress transfer pathways. This mechanism may directly reduce the modulus of rupture (MOR) and modulus of elasticity (MOE). Additionally, the observed hyphal morphology provides a critical reference for analyzing subsequent SEM images of fungal colonization within bamboo tissues.

### 3.2. Molecular Biological Identification

**Figure 4:**
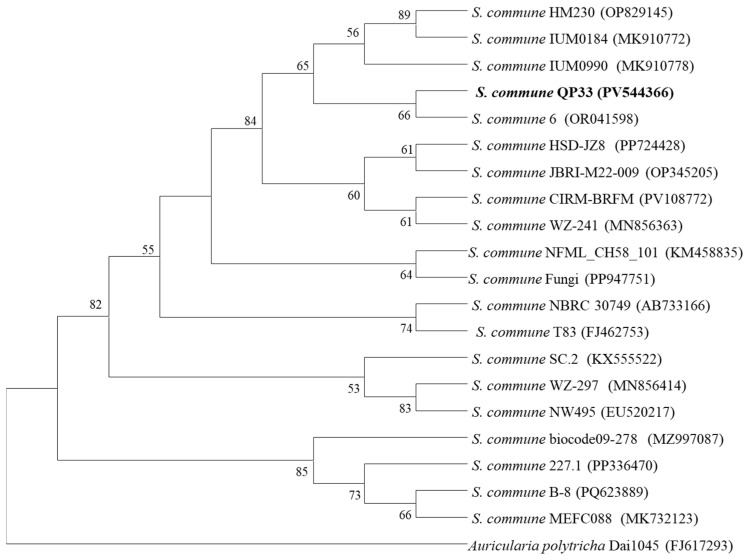
Maximum likelihood phylogenetic tree based on ITS gene sequences, showing the relationships of strain QP33 and related strains. Bootstrap values based on 1000 replicates are listed as percentages at branching points. *Auricularia polytricha* Dai1045 (FJ617293) was used as the outgroup. Only bootstrap values above 50% are shown.

The ITS sequence of strain QP33 (617 bp) was obtained with the GenBank accession number PV544366. The sequence was compared with the reported sequences in GenBank, and the results showed that the strain QP33 had high sequence similarity with members of *S. commune* (over 99%). A phylogenetic tree was constructed using the maximum likelihood method with MEGA11 (Figure 4). Phylogenetic analysis showed that the strain QP33 clustered with members of *S. commune* closely, with high bootstrap values. Based on similarity and phylogenetic analysis of ITS sequences, combined with morphological analysis, strain QP33 could be identified as a member of *S. commune*.

### 3.3. Activities of Ligninolytic Enzymes and Hemicellulases

**Figure 5:**
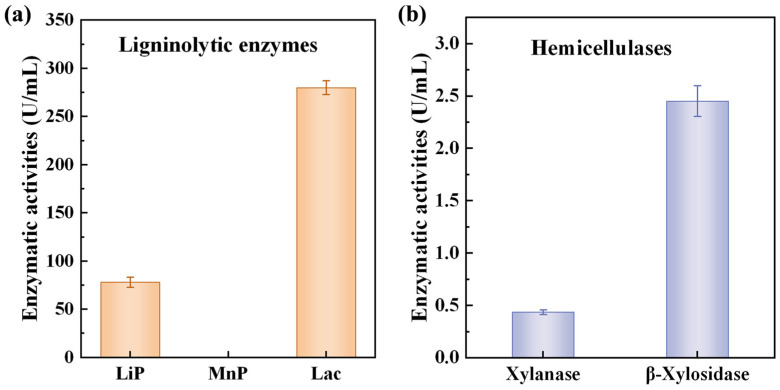
Extracellular enzyme activities of *S. commune* QP33. (**a**) Activities of lignin-modifying enzymes. (**b**) Activities of hemicellulases.

To elucidate the biochemical basis of bamboo degradation by the strain *S. commune* QP33, we measured the activities of its secreted ligninolytic enzymes and hemicellulases. As shown in Figure 5, the strain exhibited a distinct enzymatic profile. Among the ligninolytic enzymes (Figure 5a), laccase activity (280 U/mL) was significantly the highest, while no manganese peroxidase (MnP) activity was detected, and lignin peroxidase (LiP) activity (78 U/mL) was at a moderate level. This result is highly consistent with the typical enzymatic characteristics of the genus *S. commune* [15]. Genomic studies have confirmed that *S. commune* generally does not encode typical MnP and LiP genes [17], which genetically explains the low or absent activities of MnP and LiP observed in this study. Therefore, laccase is the key enzyme for lignin degradation in this strain. Laccase initiates the degradation process by oxidizing phenolic lignin units, but its ability to degrade non-phenolic structures is limited. This suggests that *S. commune* QP33 may employ a relatively “mild” lignin modification strategy, primarily aimed at “opening” the physical barrier of lignin to facilitate the subsequent degradation of hemicellulose and cellulose [39,40].

Among the hemicellulases (Figure 5b), β-xylosidase activity was significantly higher than that of endo-xylanase. This activity distribution indicates that strain QP33 may rely on other microorganisms or its own secretion of a small amount of endo-xylanase to first cleave xylan polymers into xylo-oligosaccharides. These oligosaccharides are then processed efficiently by their highly active β-xylosidase, leading to their final hydrolysis into xylose [41].

In summary, strain *S. commune* QP33 exhibits a lignocellulolytic enzyme system centered around highly active laccase and β-xylosidase. This enzymatic pattern may enable it to preferentially target the linkages in lignin-carbohydrate complexes (LCC) and efficiently utilize hemicellulose degradation products during bamboo degradation. This characteristic not only explains its ability to disrupt the cell wall structure during bamboo decay but also provides an important theoretical basis for its application in biotechnology, such as the biological pretreatment of bamboo.

### 3.4. Mass Loss and Mechanical Properties of Bamboo Infected by S. commune QP33

**Figure 6:**
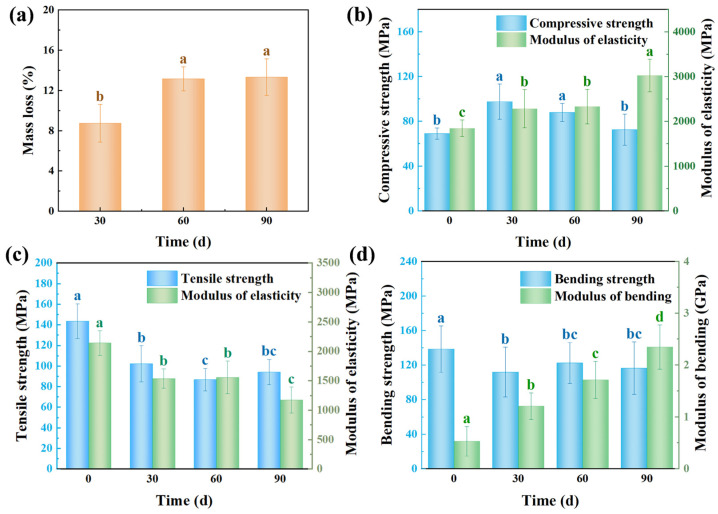
Degradation of bamboo by *S. commune* QP33 over time. (**a**) Mass loss of bamboo samples after 0, 30, 60, and 90 days of fungal decay. (**b**) Compressive strength and modulus of elasticity. (**c**) Tensile strength and modulus of elasticity. (**d**) Bending strength (modulus of rupture, MOR) and modulus of bending (MOE). Different lowercase letters (a, b, c, d) above the bars indicate significant differences among the time points according to one-way ANOVA followed by Tukey’s honest significant difference (HSD) post hoc test (*p* < 0.05).

The mass loss of bamboo infected with the white-rot fungus *S. commune* QP33 increased significantly over the 90-day incubation period, reaching 8.75% at 30 days, 13.67% at 60 days, and 13.33% at 90 days (Figure 6a, Table 1). This progressive mass loss was closely associated with the fungal secretion of lignocellulose-degrading enzymes, including laccase (Lac), lignin peroxidases (LiP), β-xylosidase and xylanase, which selectively degrade lignin and hemicellulose. The enzymatic activity may lead to the disruption of the structural integrity of bamboo by breaking down the non-cellulosic components, thereby resulting in the observed mass reduction [42,43].

Mechanical property analysis revealed distinct degradation patterns (Figure 6, Table 1). The tensile strength decreased significantly during the first 60 days (ANOVA: F (2,33) = 29.539, *p* < 0.001). This pronounced decline is attributed to the directional growth of fungal hyphae along the vascular bundles, which preferentially disrupts the integrity of longitudinal fibers, thereby critically impairing the material’s tensile resistance. In contrast, the bending strength exhibited a sharp decrease of 18.8% (from 138 MPa to 112 MPa) by day 30 but stabilized thereafter, showing no significant changes at 60 and 90 days. This stabilization suggests that while the initial fungal attack weakens the structure, the bending strength is also supported by the composite nature of bamboo, involving both longitudinal and transverse fiber architectures, which provides residual stability after the initial degradation [44,45,46]. The modulus of elasticity, however, increased significantly over time (ANOVA: F (3,33) = 2.140, *p* < 0.05). This increase, observed alongside the decline in bending strength, supports the inference that lignin degradation by *S. commune* QP33 allows for a rearrangement and compaction of the cellulose microfibrils, leading to a stiffer yet more brittle material [47]. Interestingly, the compressive strength showed a unique trend, initially increasing at 30 days, likely due to hyphal filling of cell pores, before declining significantly at 60 and 90 days (ANOVA: F = 22.880, *p* < 0.001) as substantial cell wall separation and porosity increase occurred due to extensive lignin degradation [48].

### 3.5. Ultra-Deep Field Microscope

**Figure 7:**
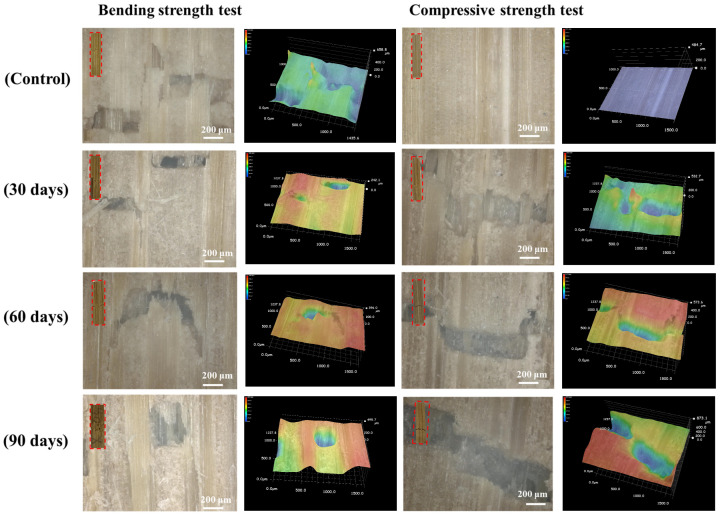
Bamboo specimen after bending and compressive strength test.

Microstructural analysis using a super depth-of-field microscope revealed the progressive deformation of bamboo following mechanical testing, correlated with the duration of fungal decay (Figure 7). The rupture severity of bamboo increased significantly with decay time under both the bending and compression tests. During the initial stages of fungal decay, hyphae primarily colonize the surface, leading to cracks that predominantly appear on the surface during fracture. During mid-late stages, hyphal penetration and hemicellulose degradation disrupted cellulose fiber continuity, leading to visible fiber fractures [49]. Compression specimens exhibited pronounced layered delamination, indicating weakened inter-wall bonding likely caused by selective lignin degradation by *S. commune*. In contrast, wood decay typically exhibits a comprehensive decline in both compressive and tensile strength. However, bamboo decay displays anisotropic damage due to its oriented fiber arrangement, where longitudinal mechanical properties (e.g., longitudinal tensile strength) are more significantly affected, while compressive strength exhibits relatively minor changes due to multiple contributing factors [3,50].

### 3.6. Microscopic Morphology Analysis of Bamboo

**Figure 8:**
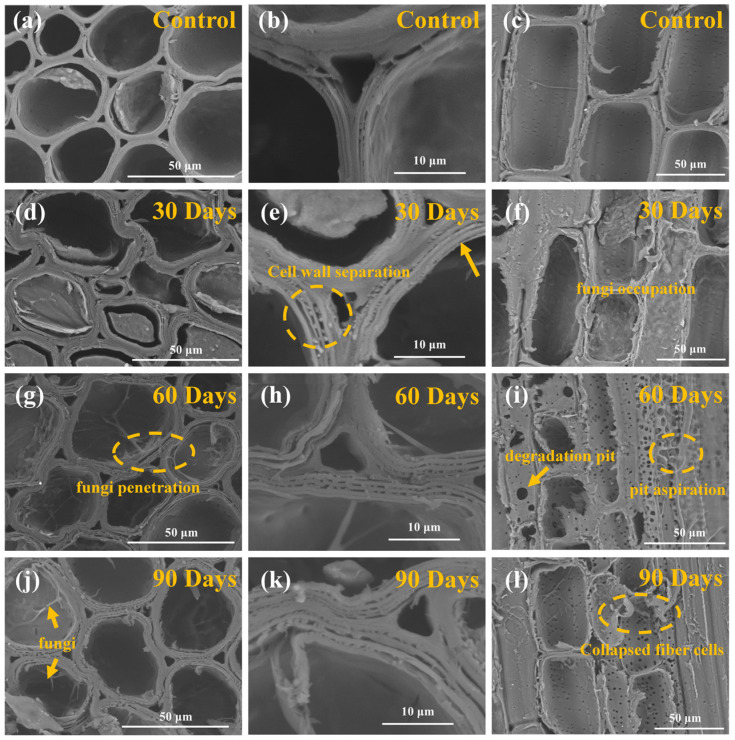
SEM photos of decayed bamboo.0 d-decayed: (**a**–**c**); 30 d-decayed: (**d**–**f**); 60 d-decayed: (**g**–**i**); 90 d-decayed: (**j**–**l**). Arrows indicate fungal hyphae, and dashed circles highlight different regions of the bamboo cell wall.

The degradation of bamboo cell walls by *S. commune* QP33 and its correlation with mechanical property loss were analyzed using SEM (Figure 8). Control samples (Figure 8a–c) showed tightly ordered, intact cell walls without stratification or mycelium. After 30 days of degradation (Figure 8d–f), pectin decomposition-initiated cell wall separation, layering, and deformation, with limited mycelial presence. Extensive microstructural degradation was observed at 60 and 90 days (Figure 8g–l), characterized by abundant mycelia, large gaps, pectin particles, and severe layering. Radial sections (Figure 8i,l) revealed numerous enlarged pores near vascular bundles, indicating profound structural damage from enzymatic pectin decomposition. These findings demonstrate that fungal enzymatic activity facilitates hyphal penetration, leading to pore enlargement, microstructural deterioration, and the consequent significant reduction in mechanical properties [1,51,52].

### 3.7. FTIR Analysis

**Figure 9:**
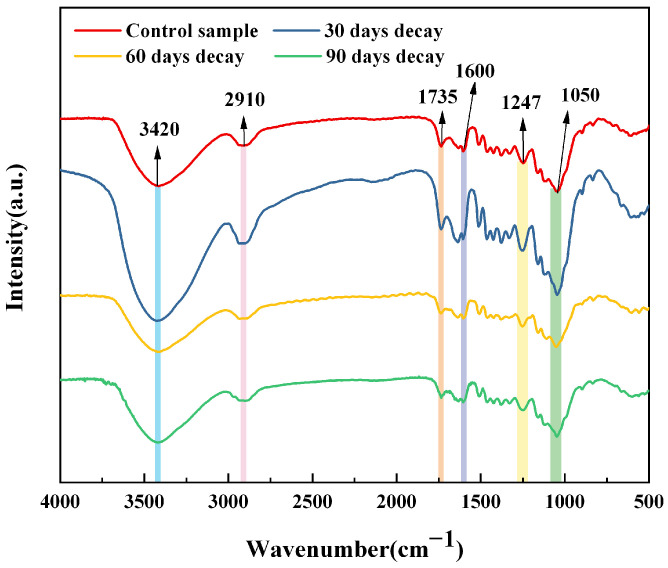
FTIR spectra of bamboo timber after decaying at different times.

In order to better analyze the damage to the chemical composition of bamboo during the degradation process of *S. commune* QP33, FTIR was applied and the results are shown in Figure 9. 1600 cm^−1^ mainly represents the carbon skeleton vibration of the benzene ring in lignin, 3420 cm^−1^ represents the change of hydroxyl group, 1735 cm^−1^ is the telescopic vibration of carbonyl group, which is mainly the change of xyloglucan acetyl group [53]. The change of 1247 cm^−1^ was caused by stretching vibrations of the benzene ring-oxygen bond in lignin, and 1050 cm^−1^ represented stretching vibrations of C-O, mainly from cellulose and hemicellulose [54,55,56]. The absorption peaks at 1600 cm^−1^, 1050 cm^−1^, and 1247 cm^−1^ for lignin, 1735 cm^−1^ for hemicellulose and 1050 cm^−1^ for cellulose were significantly shifted and weakened in the samples after degradation with *S. commune* QP33, indicating that *S. commune* QP33 mainly degraded lignin and hemicellulose.

### 3.8. XPS and XRD Analysis

**Figure 10:**
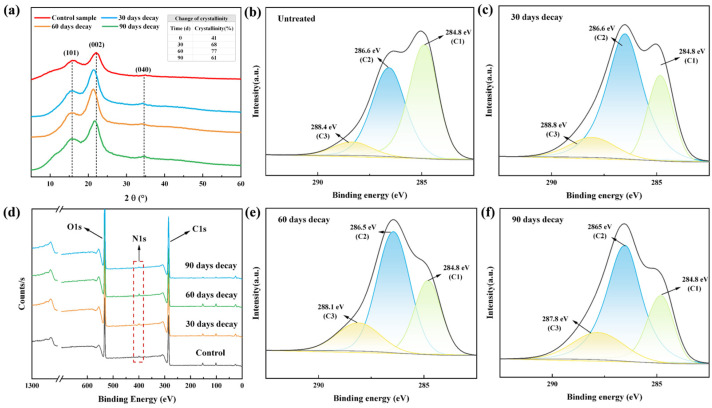
Evolution of crystalline structure and chemistry of bamboo during decay by *S. commune* QP33. (**a**) XRD patterns of bamboo at different decay stages (control, 30 days decay, 60 days decay, and 90 days decay). The table in the inset shows the change in crystallinity with decay time. (**b**–**f**) XPS spectra of bamboo samples at different decay stages, including survey spectra and high-resolution C1s spectra.

To elucidate the degradation mechanism of bamboo by *S. commune* QP33, the chemico-microstructural evolution was examined via X-ray photoelectron spectroscopy (XPS) and X-ray diffraction (XRD). These structural changes were then correlated with the decline in macroscopic mechanical properties (Figure 10) [44]. The C1s spectrum from XPS was deconvoluted into three primary components: the C1 peak (C-C/C-H), representing aromatic rings and aliphatic side chains in lignin and extractives; the C2 peak (C-O), indicative of hydroxyl and ether bonds in cellulose and hemicellulose; and the C3 peak (C=O/O-C-O), assigned to carbonyl groups in hemicellulose and aldehyde groups at cellulose chain ends [53].

During initial decay (30 days), a sharp decrease in C1 content and an increase in C2 indicated preferential degradation of lignin and hemicellulose [57]. This selective removal of amorphous matrix components led to increased cellulose crystallinity as shown by XRD (Figure 10), contrasting sharply with the crystallinity decrease characteristic of brown-rot fungi [38]. The loss of interfacial bonding from matrix degradation directly caused the initial 18.8% reduction in bending strength [58,59]. A critical transition occurred at mid-stage (60 days), where C2 content decreased significantly, revealing fungal attack expanding to cellulose microfibrils, the primary load-bearing skeleton. This structural failure resulted in the most severe deterioration of tensile and compressive strength (Figure 6b,c). The concurrent O/C ratio increased to 69.5% (as shown in Table 2), indicating accumulation of oxygen-rich degradation products. By the final stage (90 days), the relative contents of both C1 and C2 components changed drastically again. The O/C ratio dropped further to 53%, suggesting the possible carbonization of degradation residues [60]. A sharp decrease in nitrogen content was also detected, likely attributable to the release and loss of protein-rich fungal mycelia as the bamboo substrate was thoroughly decomposed.

The dynamic evolution of the bamboo surface chemistry, as revealed by XPS (e.g., decrease in C1 component, fluctuation in O/C ratio), confirms the oxidative action of fungal enzymes. However, the extent of these changes appeared less pronounced than that typically caused by white-rot fungi possessing robust peroxidase systems (e.g., LiP, MnP), such as *Trametes versicolor* [10]. This can be attributed to the unique enzymatic profile of *S. commune* QP33, which is dominated by laccase with minimal peroxidase activity. This profile results in a relatively milder and more selective surface modification and degradation strategy, ultimately leading to the distinctive, multi-stage deterioration pattern of mechanical properties observed in this study.

## 4. Conclusions

This study systematically elucidates the spatiotemporal degradation mechanism of bamboo by the white-rot fungus *S. commune* QP33. The results demonstrate that the strain employs a selective lignocellulose degradation strategy, primarily driven by a unique enzymatic system dominated by laccase. Over the 90-day decay period, bamboo exhibited a progressive mass loss, reaching 13.33%, accompanied by distinct temporal deterioration patterns in its mechanical properties. The bending strength decreased sharply by 18.8% within the initial 30 days before stabilizing; the tensile strength declined gradually over time, while the compressive strength showed a unique trend of an initial increase, likely due to hyphal filling of voids, followed by a significant decrease as substantial structural damage occurred. Microstructural and chemical analyses revealed that the fungal hyphae preferentially colonized and degraded the amorphous matrix components, primarily lignin and hemicellulose. This selective degradation led to the separation of the inter-fiber layer and delamination of the cell wall, which subsequently triggered the macroscopic deterioration of mechanical properties. FTIR and XPS analyses confirmed the enzymatic oxidation process, evidenced by the attenuation of the characteristic lignin peak (1600 cm^−1^) and an increase in surface C=O groups. XRD results indicated a rise in cellulose crystallinity as the degradation progressed, reflecting the relative enrichment of crystalline cellulose following the selective consumption of amorphous components. These comprehensive findings clarify the degradation mode of *S. commune*, which initiates by attacking the non-crystalline regions, thereby compromising the overall structural integrity of bamboo. This study not only provides critical experimental evidence and a theoretical framework for understanding the interactions between white-rot fungi and bamboo but also establishes a scientific foundation for future green biotechnology applications utilizing *S. commune*, such as the biological modification of bamboo and the resource utilization of waste biomass.

## Figures and Tables

**Table 1:** One-way ANOVA results of mass loss and mechanical properties of bamboo infected by *S. commune* QP33 for different durations.

Dependent Variable	F-Value	*p*-Value
Mass loss	29.54	0.00
Tensile strength (MPa)	29.54	0.00
Modulus of elasticity (MPa)	29.54	0.00
Bending strength (MPa)	2.14	0.11
Modulus of bending (GPa)	36.52	0.00
Compressive strength (MPa)	22.88	0.00
Modulus of elasticity (MPa)	3.36	0.04

**Table 2:** Atomic percentages of carbon and oxygen at different times.

Atomic %							
Time (d)	C1(C-C/C-H)	C2(C-O)	C3(C=O/O-C-O)	C1s	O1s	N1s	O/C
0	23.95	31.59	4.40	59.95	38.96	1.09	65
30	16.65	38.19	7.11	61.97	36.82	1.21	59.4
60	9.58	18.73	5.66	58.31	40.55	1.14	69.5
90	15.96	37.57	11.58	65.11	34.48	0.41	53

## Data Availability

Data will be made available on request.
